# A Promising 1,3,5-Triazine-Based Anion Exchanger for Perrhenate Binding: Crystal Structures of Its Chloride, Nitrate and Perrhenate Salts

**DOI:** 10.3390/molecules28041941

**Published:** 2023-02-17

**Authors:** Valery N. Zakharov, Pavel S. Lemport, Vladimir V. Chernyshev, Victor A. Tafeenko, Alexandr V. Yatsenko, Yuri A. Ustynyuk, Sergey F. Dunaev, Valentine G. Nenajdenko, Leonid A. Aslanov

**Affiliations:** Department of Chemistry, Lomonosov Moscow State University, Leninskie Gory 1 bld. 3, Moscow 119991, Russia

**Keywords:** 1,3,5-triazine, anion exchanger, precipitation of perrhenate, crystal structure, NMR, DFT

## Abstract

The reaction of pyridine with cyanuric chloride was studied under microwave activation as well as in the presence of silver nitrate. The product of hydrolysis containing two pyridinium rings and chloride anion was isolated. The structures of these anion exchanger salts with chloride, nitrate and perrhenate anions are discussed.

## 1. Introduction

Technetium-99 (^99^Tc, β-emitter 0.2938 MeV) is formed during artificial nuclear fission decay of ^235^U in nuclear reactors. Each light water nuclear reactor produces about 2 g of ^99^Tc for every 100 MW of thermal energy produced, thus yielding about 20 kg per year. The total amount of ^99^Tc in the spent nuclear fuel (SNF) of all nuclear power plant reactors in the world accumulated per year is close to 10 tons. During SNF processing, technetium is stabilized as the pertechnetate anion TcO_4^−^_. The high solubility of alkali metal pertechnetates in water (11.3 mol/L at 20 °C for NaTcO_4_) and their low sorption on rocky and salt mineral rocks determine the high mobility of TcO_4^−^_ in groundwater and in near-surface layers of the earth crust. In combination with a long half-life (t_1/2_ = 2.13 × 10^5^ years) it makes TcO_4^−^_ one of the most environmentally hazardous radioactive pollutants when released into the environment [1,2]. Therefore, the separation of ^99^Tc from SNF is a task of paramount importance.

At present, the PUREX process is the main SNF reprocessing technology. SNF is kept for a period necessary for the decay of most short-lived radionuclides and after that is dissolved in nitric acid. In the first cycle of the PUREX process, uranium and plutonium are extracted from acidic aqueous solutions with a 30% solution of tributyl phosphate (TBP) in a hydrocarbon solvent in the form of complexes [UO_2_(NO_3_)_2_(TBP)_2_] and [Pu(NO_3_)_4_(TBP)_2_]. However, 30–90% of technetium (depending on the extraction conditions) passes into the organic phase mainly in the form of a mixed complex [UO_2_(NO_3_)(TcO_4_)(TBP)_2_]. The presence of TcO_4^−^_ in the organic phase significantly complicates the reductive re-extraction of plutonium. Technetium is able to be reduced to the oxidation states +4 and +5. As a result, it acts as a catalyst for the decomposition of hydrazine used as a reducing agent and catalyzes the oxidation of Pu^3+^ with nitric acid. The presence of pertechnetate ions also significantly complicates the final process of SNF vitrification. This technological step is carried out at high temperatures. Technetium can form various volatile compounds (Tc_2_O_7_, HTcO_4_ and others) to be captured. Only a small part of technetium is included in the borosilicate matrix. According to the International Atomic Energy Agency, by the end of 2020 about 250,000 tons of SNF was accumulated. The difficulties of such processing within the PUREX technology are obvious: high acidity (3M HNO_3_), high ionic strength (large amounts of metal and nonmetal ions), and strong ionizing radiation field. To exclude the possibility of ^99^Tc leakage into the environment, it is necessary to extract ^99^Tc at the initial stage of their processing, which is a complex and yet unsolved problem. Research aimed at the creation of efficient technologies for the separation of ^99^Tc from SNF have been intensively developed, especially in the last decade. Various methods for the selective binding of TcO_4^−^_ have been proposed: precipitation from solution in the form of insoluble salts; isolation via anion exchange reactions with ion exchange resins and various inorganic materials, organic and organometallic frameworks; Tc binding by its immobilization on inorganic matrices with partial reduction; isolation via extraction of TcO_4^−^_ with highly selective organic receptors in two-phase systems (organic solvent–aqueous solution), as well as other methods. Each method has some advantages and disadvantages but none of them are a universal one [3,4,5,6,7,8,9,10].

The precipitation of TcO_4^−^_ from aqueous solutions in the form of insoluble salts is one of the most efficient and cost-effective isolation methods, which does not require the use of organic solvents. Quaternary phosphonium and arsonium salts [11,12,13,14], guanidinium salts [15] and cationic 1,2,4-selenodiazoles [16] have been used as precipitants. The most promising results have been obtained using polycations [17].

However, the high complexity of synthesis and high cost are the main disadvantages of these methods. In this regard, the development of other polycationic receptors seems to be a promising way to solve the problem of TcO_4^−^_ precipitation from SNF solutions.

Cyanuric chloride C_3_N_3_Cl_3_ is industrially available and a low-cost triazine. This compound is also a highly reactive electrophile. Its reactions with nucleophiles are well documented to open the accessibility to various triazine derivatives. 1,3,5-Triazines have been actively studied in the design of biologically important organic substances [18,19] with antimicrobial [20], antimalarial [21], antifungal [22], analgesic [23], antitumor [24,25], and antistaphylococcal [26] properties. There is a lot of literature on the synthesis of triazine 2D covalent organic frameworks (COFs) [27], which have the properties of sorbents, catalysts and photocatalysts, materials for battery and supercapacitor electrodes, membranes, and used in optoelectronics. Some of the triazine backbones are derived from cyanuric chloride.

We proposed that the reaction of pyridine with cyanuric chloride could open the accessibility to polycationic salts possessing a high anion capacity (up to three anions to one molecule). An efficient molecular weight per one anion is as low as 104 if three pyridine molecules enter the reaction with cyanuric chloride. Moreover, we expected that such a straightforward approach would be very simple and cost efficient. To our surprise, products of the reaction of pyridine with cyanuric chloride have not been studied in detail. This system (pyridine–cyanuric chloride) has been used for the activation of carboxylic acids [28,29,30] under mild conditions. Nucleophilic substitution of chloride ions in cyanuric chloride with 4,4’-dipyridyl has been used for the synthesis of COFs [31]. However, due to the high electron deficiency of the triazine ring, the prepared COFs undergo partial reduction by chloride ions to form free radical species with concentration 1 × 10^20^ spin g^−1^. Thus, the reaction of cyanuric chloride with pyridine and its derivatives requires further study. 

In this work, we investigated the reaction of pyridine and cyanuric chloride for the subsequent binding of anions. We expected that such a system could be efficient for pertechnetate anions. We used perrhenate anions as a model. It is known that these two anions are very similar in geometry as well as chemical properties [32,33,34].

## 2. Results and Discussions

### 2.1. Synthesis and X-ray Phase Analysis

**Scheme 1:**
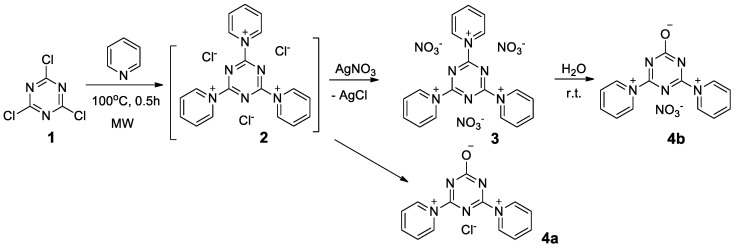
Reaction of cyanuric chloride with pyridine in the presence of AgNO_3_.

We started our study by investigating the reaction of pyridine with cyanuric chloride **1** (Scheme 1). Surprisingly, a model reaction of cyanuric chloride with significant access of pyridine gave no product at 30 °C during a 5 min reaction. However, a black precipitate was formed when microwave activation was used in a CEM microwave reactor. After heating for 5 min, an excess of pyridine was decanted, and the precipitate was washed with dichloromethane and dried. The powder diffraction pattern of the obtained precipitate is shown in Figure S1a (Supplementary Materials). No cyanuric chloride was detected; however, a mixture of products was obtained. We believe that the deep black color of the precipitate is due to electron transfer from pyridine ligands to the triazine ring with the formation of radical species as was reported previously for the reaction of cyanuric chloride with bipyridine [31]. In subsequent experiments, the reaction conditions remained almost identical, but the reaction time was increased to 10, 20 and 30 min. The X-ray patterns of the precipitates are shown in Figure S1b–d, respectively. A comparison of X-ray patterns indicates that the reaction of cyanuric chloride with pyridine proceeded. However, all reaction conditions provided a mixture of products.

It is known that silver salts can be used for the activation of halogen derivatives in substitution reactions. A nearly single product **4a** was obtained when 6 mmol of AgNO_3_ was dissolved in 4 mL of pyridine and the prepared solution was added to 2 mmol of cyanuric chloride in a glove box. The mixture was stirred for 10 min at 27 °C. A brown precipitate (a mixture of AgCl and the intermediate product) was formed. Pyridine was decanted and the precipitate was washed with ethanol to remove traces of pyridine. The reaction product of cyanuric chloride with pyridine was washed with water (product was found to be insoluble in ethanol) and the product was precipitated from an aqueous solution using 80 vol.% of diglyme. The dark brown precipitate was centrifuged and dried (yield 21%). We expected to obtain product **2** with three pyridine rings in the structure. Surprisingly, the chemical composition of the crystals determined unambiguously by X-ray diffraction analysis gave us the structure [(C_3_N_3_O)(C_5_H_5_N)_2_]Cl·H_2_O. This compound seemed to be a product of the hydrolysis of intermediate **2** bearing three pyridinium rings. We believe that due to the high concentration of the positive charge in molecule **2,** Coulomb repulsion facilitated hydrolysis of one pyridinium moiety. As a result, compound **4a** bearing two pyridinium ring was isolated. One additional driving force of such transformation can be pointed out: the second positive charge in structure **4a** was compensated by deprotonation. The product **4a** contained about 10% impurities of product **4b** (see below), a result of partial anion exchange. Thus, cyanuric chloride actively reacted with pyridine in a microwave reactor at 100 °C. The reaction proceeded more smoothly without a microwave reactor, but activation with silver nitrate was required.

We decided also to selectively prepare the corresponding product **4b**. For this, 6 mmol of AgNO_3_ was dissolved in 4 mL of pyridine and then cyanuric chloride (2 mmol) was added to the resulting solution in a glove box. The mixture was stirred under reflux during 60 min at 100 °C to form a brown precipitate. Pyridine was decanted and the precipitate was treated with water with vigorous shaking. Next, the mixture was centrifuged to separate the solution of the resulting compound from solid silver chloride. Then, 20 mL of diglyme was added to 5 mL of the resulting aqueous solution. The flaky white precipitate was separated in a centrifuge, washed with ethanol and dried at 130 °C in air (yield 29%). Chemical composition of crystals [(C_3_N_3_O)(C_5_H_5_N)_2_]NO_3_ was determined by X-ray diffraction analysis. The crystal structure of the compound [(C_3_N_3_O)(C_5_H_5_N)_2_]NO_3_ is described below. Therefore, prolonged heating was required for complete anion exchange to transform **4a** in the corresponding nitrate **4b**.

**Scheme 2:**
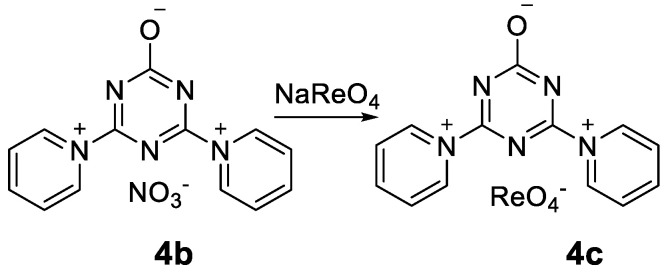
Precipitation of perrhenate **4c**.

The perrhenate anion ReO_4^−^_, with the solubility of its salts coinciding with that of the corresponding pertechnetate salts, is often used in experiments as a non-radioactive model of the pertechnetate anion. Obtaining **4b** we studied its reaction with sodium perrhenate. For this, the freshly obtained aqueous solution of **4b** was used. The reaction of cyanuric chloride with pyridine was performed in the presence of silver nitrate under the abovementioned conditions. Next, 18 mL of water was added to extract the formed product **4b** (0.58 mmol). The obtained clear solution was mixed with an aqueous solution of sodium perrhenate (6 mmol of NaReO_4_ in 10 mL of water). Nearly a 10-fold excess of NaReO_4_ was used for the isolation of a new product **4c** with unknown solubility (Scheme 2).

Relatively large brown crystals of **4c** were formed with a 79% yield. The solubility of the product in water was 1 g per 100 mL. X-ray diffraction confirmed the [(C_3_N_3_O)(C_5_H_5_N)_2_]ReO_4_·3H_2_O composition of the formed crystals. 

NMR spectra of **4a–c** are very similar. For example, the ^1^H NMR spectrum of compound **4b** (Figure S2) is in agreement with the structure of this compound. Proton signals of the quaternized pyridinium substituents were observed in the aromatic part. Their chemical shifts, relative intensity and multiplicity correspond with the structure of **4b**. In the ^13^C NMR spectrum of **4b**, signals with chemical shifts of 169.5 and 164.0 ppm were observed, corresponding to the carbon atoms of the triazine ring. The IR spectrum was recorded for compound **4c** (Figure S3). The spectrum confirmed the presence of the perrhenate anion in the molecule, appearing as a characteristic band at 898 cm^−1^, slightly shifted from the corresponding band of sodium perrhenate (888 cm^−1^). In addition, a mass-spectrometry study of **4b** was performed to demonstrate the highly intensive peak at 252.1 Daltons (Figure S4), corresponding to 4,6-dipyridinio-2-oxido-1,3,5-triazinecation.

**Figure 1:**
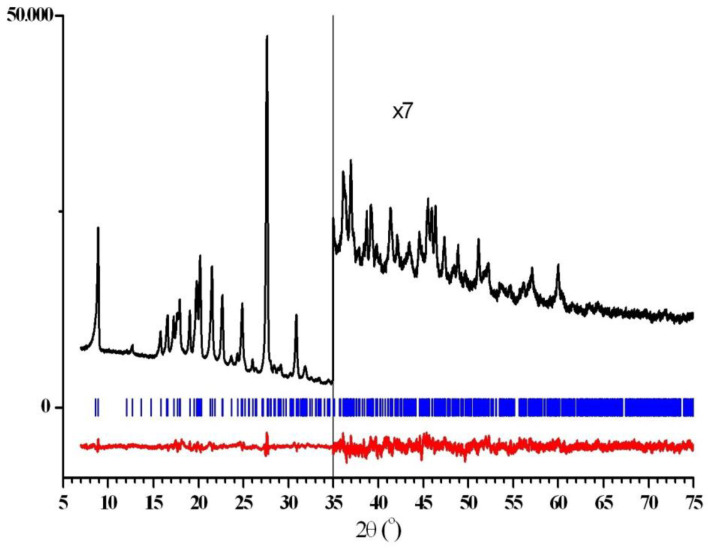
The final Rietveld plot for **4b** showing the experimental diffraction profile in black and the difference (exp–calc) profile in red. The vertical blue bars correspond to the calculated positions of the Bragg peaks.

**Figure 2:**
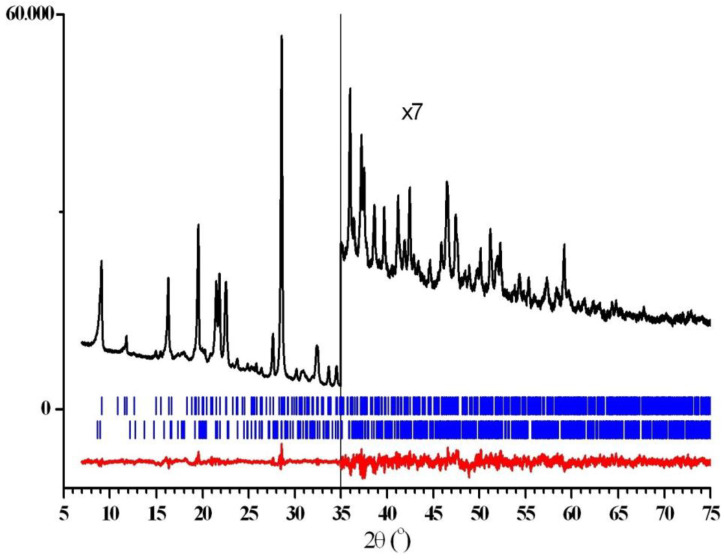
The final Rietveld plot for **4a** after the final two-phase refinement showing the experimental diffraction profile in black and the difference (exp–calc) profile in red. The vertical blue bars correspond to the calculated positions of the Bragg peaks for two crystalline phases—**4a** (1st raw) and **4b** (2nd raw).

For the two compounds **4a** and **4b**, whose structures were determined from the X-ray powder data, the experimental and different diffraction profiles after final bond-restrained Rietveld refinements are shown in Figure 1 and Figure 2, respectively. The crystal data, data collection and refinement parameters for both compounds are given in Table S1.

**Figure 3:**
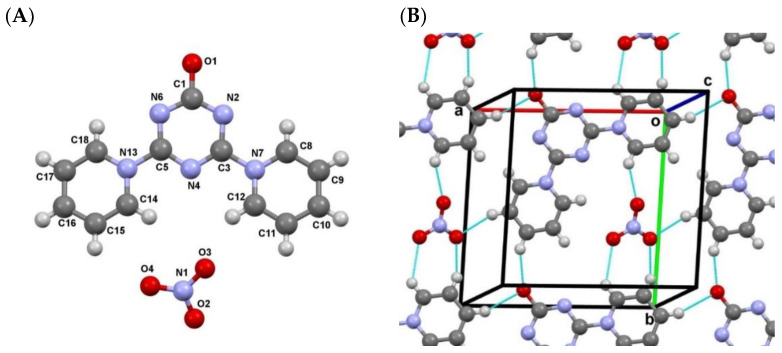
(**A**) Content of the asymmetric unit of **4b** showing the atomic numbering; (**B**) a portion of the crystal packing of **4b** showing the intermolecular C-H…O hydrogen bonds (cyan thin lines), which link the cations and anions into sheets parallel to the *ab* planes.

The molecular structure and portion of the crystal packing of **4b** are shown in Figure 3A and B, respectively, prepared with the *Mercury* program [35]. Weak intermolecular C-H…O hydrogen bonds (Table S2) link all moieties into sheets parallel to the *ab* plane (Figure 3B). The short intermolecular contacts between the negatively (O1, O3 from NO_3_ anion) and positively (N7, N13) charged atoms—O1…N7(1 − *x*, −*y*, −*z*) 3.078(7) Å, O3…N13(1 − *x*, 1 − *y*, 1 − *z*) 2.989(8) Å—reveal dipole–dipole interactions between the neighboring sheets (Figure S5), which further consolidate the crystal packing.

**Figure 4:**
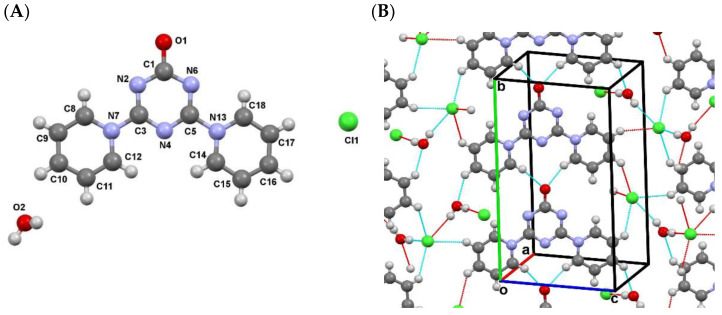
(**A**) Content of the asymmetric unit of **4a** showing the atomic numbering; (**B**) crystal packing of **4a** showing the intermolecular O-H…Cl, C-H…Cl and C-H…O hydrogen bonds as thin cyan and red lines.

The molecular structure and portion of the crystal packing of **4a** are shown in Figure 4A,B. The intermolecular O-H…Cl, C-H…Cl and C-H…O hydrogen bonds (Table S3) link all moieties into the 3D crystal structure (Figure 4B). The crystal packing also exhibits short intermolecular contacts between the negatively (O1, Cl1^−^) and positively (N7, N13) charged atoms (Figure S6), which also contribute to the crystal packing consolidation, although they are much weaker compared with those in **4a–**O1…N13 (*x*, 3/2 − *y*, −1/2 + *z*) 3.262(9) Å, Cl1…N7(1 + *x*, *y*, 1 + *z*) 3.394(8) Å.

**Figure 5:**
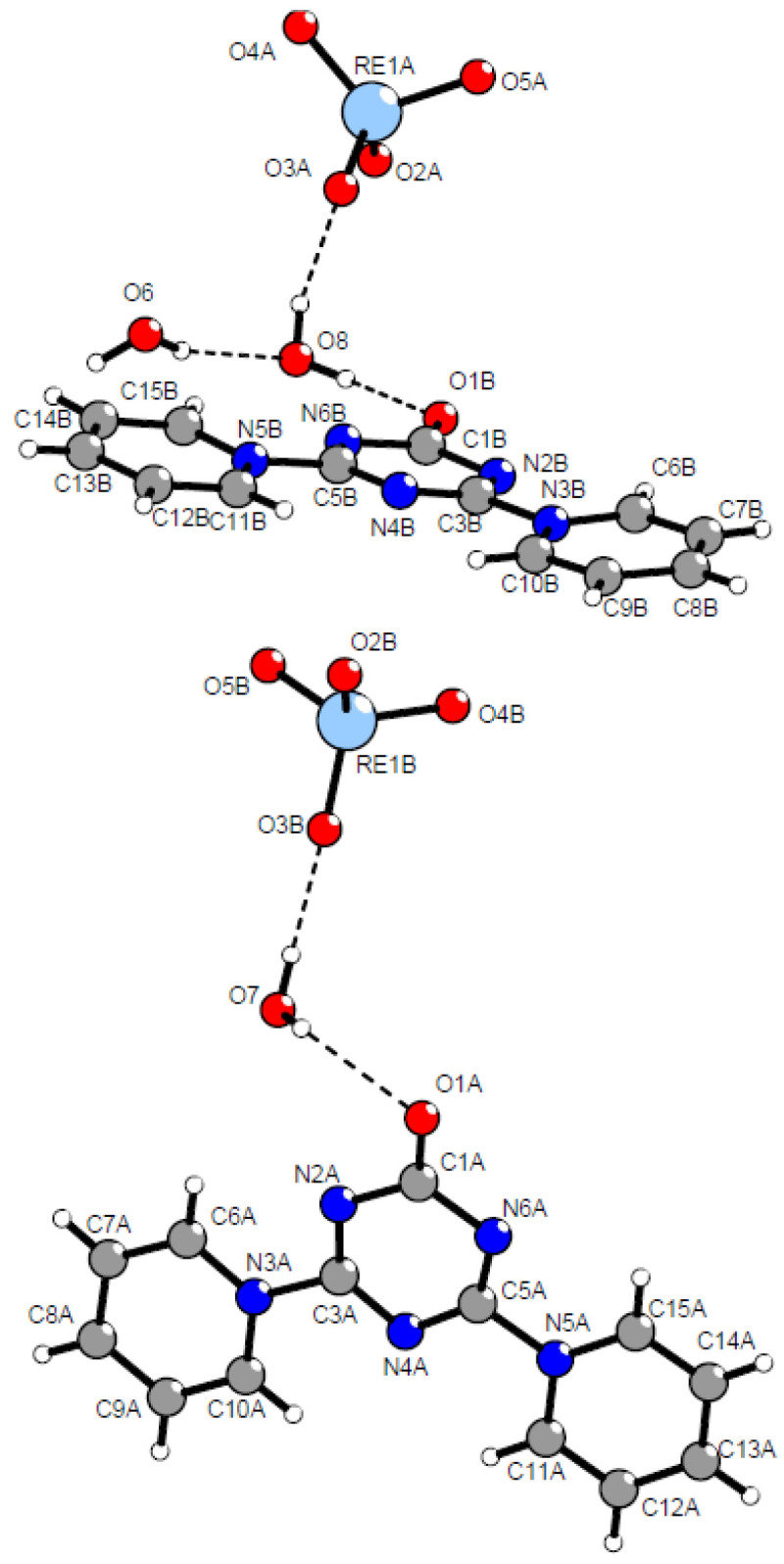
Numeration of atoms in the **4c** ions. Displacement ellipsoids are drawn at a 30% probability level.

The crystal structure of 4,6-dipyridinio-2-oxido-1,3,5-triazine perrhenate **4c** is shown in Figure 5, while Figure S7 demonstrates the hydrogen bonds in the **4c** crystals. Table S4 presents the values of hydrogen bonds.

Two independent cations and two perrhenate ions are joined by hydrogen bonds via three water molecules. The observed systematic intergrowth of perrhenate crystals complicates the analysis of the electron density distribution pattern, namely, two unidentified maxima are located at distances of ~2.4 Å from each rhenium atom. They are not strong, but noticeable. Their position relative to the oxygen and metal atoms of perrhenate ions makes their interpretation rather complicated. However, their presence does not raise doubt in the correctness of the given crystal structure. The chemical composition of the compound was determined from the results of X-ray diffraction analysis.

### 2.2. Mutual Arrangement of Cations and Anions in the Structures ***4a***, ***4b*** and ***4c***

**Figure 6:**
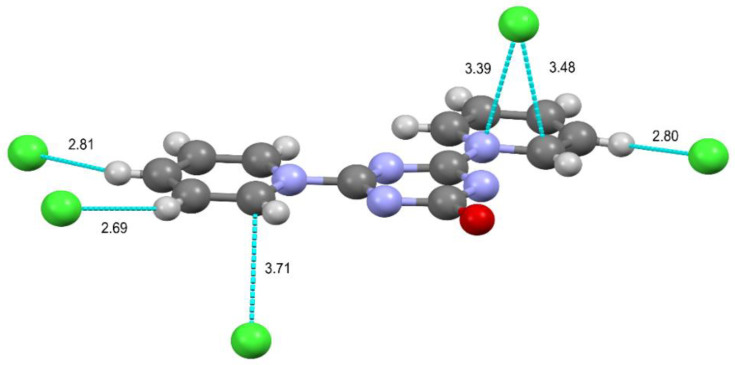
Cation in **4a** surrounded by chloride anions and the shortest C-H...Cl, C...Cl and N...Cl contacts (Å).

In the hydrated structures, the extended systems of hydrogen bonds involving water molecules are formed, but here we shall only consider the mutual arrangement of cations and anions and the contacts between them. In **4a** each cation is surrounded by five Cl^–^ anions. Three of them are situated near the main plane of the cation and form weak hydrogen bonds C–H...Cl with it, whereas two others lie on both sides of this plane (Figure 6). The contacts Cl...H are slightly shorter than the sum of van der Waals radii (2.95 Å), whereas the out-of-plane contacts C...Cl and N...Cl are longer than the corresponding reference values (3.30 Å for Cl...N and 3.45 for Cl...C).

**Figure 7:**
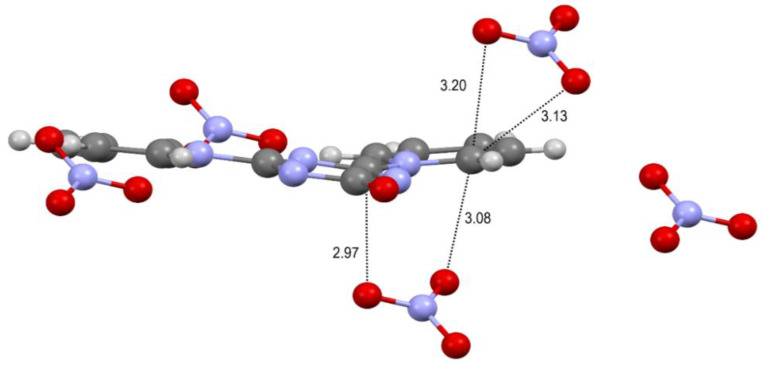
A cation surrounded by nitrate ions in the **4b** structure, with three nitrates in the same layer and two from the adjacent layers.

In the structure **4b**, nitrate ions link cations into layers (Figure 3B), and the O...H distances are noticeably shorter than the sum of van der Waals radii (2.72 Å). Additionally, the aromatic systems of cations partly overlap with nitrates of the adjacent layers. The cation surrounded by three nitrate ions of the same layer and two nitrates above and below is presented in Figure 7. The shortest O...C contacts are close to the sum of van der Waals radii (3.22 Å). The nitrate ions are bent to the cation mean plane, so each of them forms only two O...C contacts.

**Figure 8:**
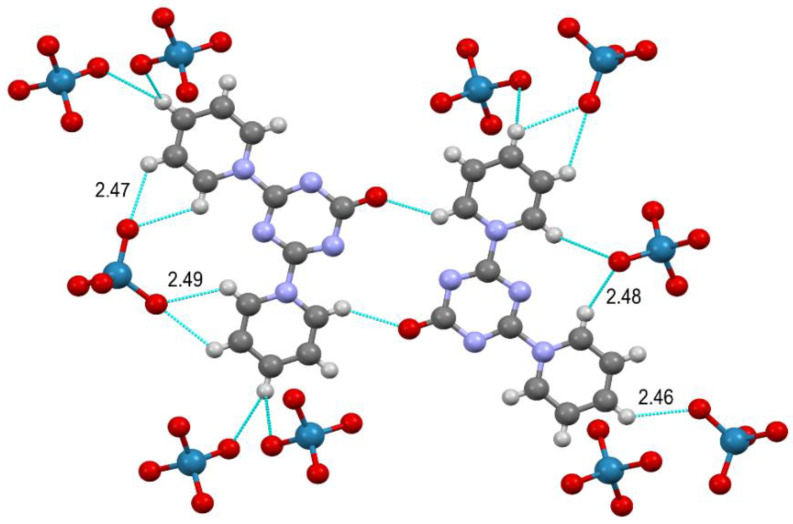
Dimer formed by cations linked by C-H…O contacts in the **4c** structure. All the contacts with perrhenate ions which are not designated are longer than 2.50 Å.

In the **4c** structure (Figure 5), there are two crystallographically non-equivalent cations denoted as A and B and two anions. Figure 8 demonstrates how cations are linked into dimers by hydrogen bonding, and how the periphery of this dimer is decorated by perrhenate ions. These ions form the C-H...O contacts with cations, but they are noticeably longer than analogous contacts in the structure of nitrate salts.

**Figure 9:**
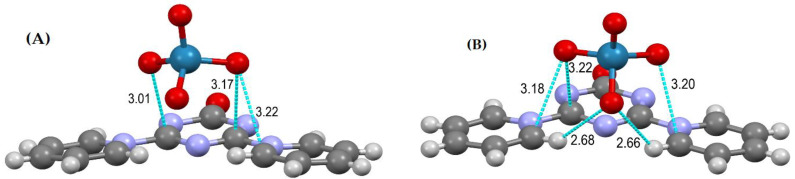
Ion pairs formed by (**A**,**B**) cations with perrhenate anions.

Perrhenate ions form more significant contacts with the π-systems of cations. The structures of both ion pairs formed by A and B cations are very much alike (Figure 9). A negligible difference is that in the ion pair with cation B perrhenate is slightly bent, and due to this one of its O atoms form the O...H contacts, but the O...C contacts of two other O atoms appear to be slightly longer than in the other ion pair. The architectures of ion pairs in crystal structure generally match with that optimized by DFT.

### 2.3. DFT Calculations

**Figure 10:**
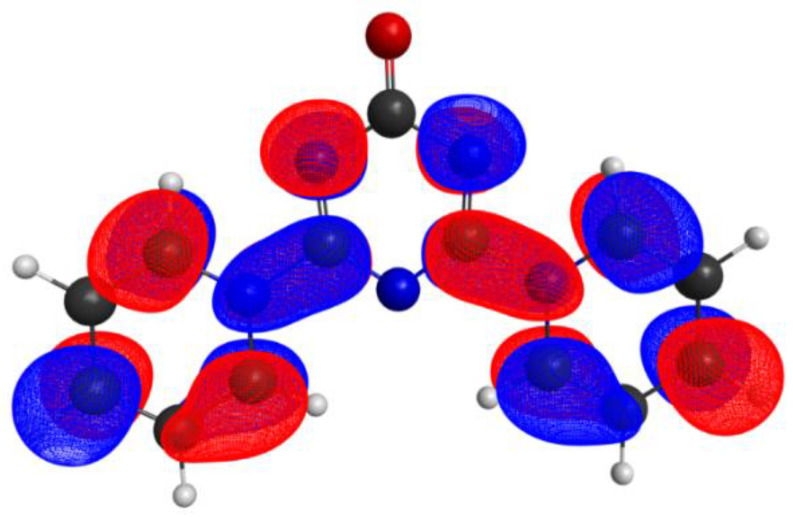
4,6-Dipyridinio-2-oxido-1,3,5-triazine perrhenate: its LUMO is a π-type orbital delocalized over almost the entire cation.

To gain insight into the obtained results, we performed DFT calculations. The calculation of the cation showed that its LUMO is almost uniformly delocalized over all three aromatic rings (Figure 10), so that the nucleophile (anion) can attach to the cation at any point on its surface.

**Figure 11:**
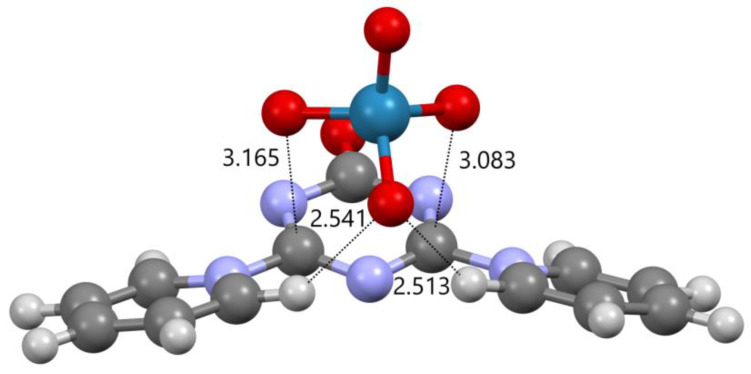
4,6-Dipyridinio-2-oxido-1,3,5-triazine perrhenate: the shortest anion–cation contacts (Å) are indicated by dotted lines.

It was also found that the structure shown in Figure 11 corresponds to the energy minimum of the ion pair. Two oxygen atoms of the perrhenate anion form contact with carbon atoms of the triazine ring, and the third oxygen atom forms contacts with α-pyridinium hydrogen atoms. All contacts are close in length to the sum of the van der Waals radii. 

## 3. Materials and Methods

### 3.1. Materials

The following reagents were used in this work: pyridine (special purity), which was additionally distilled and stored over A4 zeolite; diethylene glycol dimethyl ether (diglyme); dichloromethane; silver nitrate (chemically pure); cyanuric chloride (98%); sodium perrhenate (pure); and ethanol rectified were all purchased from Sigma Aldrich (St. Louis, MO, USA). Deuterated solvents for NMR spectra registration were purchased from Cambridge Isotope Laboratories, Inc. (Andover, MA, USA) and used without further purification.

### 3.2. Synthesis Conditions

The CEM Focused Microwave TM synthesis system, model Discover (USA), was used to perform chemical reactions under atmospheric pressure. For work with hygroscopic compounds, a sealed vacuum glove box SPECS GBVK (Russia) with a control unit and one gateway was used, the pressure during pumping was 10^−2^ mm Hg. A Multi Centrifuge CM 6M (Elmi Ltd., Rīga, Latvia) 3500 rpm was used to separate the precipitates. 

### 3.3. X-ray Analysis

#### 3.3.1. Powder Diffraction

Powder patterns were measured using two powder diffractometers, namely, Empyrean (Cu K_α_ radiation, Ni β-filter, Bragg−Brentano mode) and Huber G670 Guinier camera (Cu K_α1_ radiation, λ = 1.54059 Å, transmission mode). Empyrean was used for high-throughput preliminary measurements of all synthesized samples in a low-angle range 5–40°. The crystal structures of **4a** and **4b** (see above) were established with the powder patterns measured using the Huber G670 Guinier camera (see Table S1 for data collection details). Crystal structures were solved with the use of a simulated annealing technique [36] and refined with the program MRIA [37] in the same way as described by us previously [38,39].

#### 3.3.2. Single-Crystal Diffraction

The data were collected at room temperature by using an STOE diffractometer Pilatus 100K detector, focusing mirror collimation Cu K_α_ (1.54086 Å) radiation, in rotation method mode. The STOE X-AREA software was used for cell refinement and data reduction. Data collection and image processing was performed with X-Area 1.67 (STOE & Cie GmbH, Darmstadt, Germany, 2013). Intensity data were scaled with LANA (part of X-Area) in order to minimize the difference in intensities of the symmetry-equivalent reflections (multi-scan method). The structure was solved with SHELXT and refined with the SHELX program [40]. There was an intergrowth of crystals in **4c** (see above), evidenced by the obtained results; for example, the accuracy of bond lengths were not higher than 0.01–0.02 Å and the appearance of significant but non-interpretable peaks on the electron density difference maps. About a dozen samples were tested before a satisfactory crystal was found. The non-hydrogen atoms were refined by using the anisotropic full matrix least-square procedure. All hydrogen atoms were placed in the calculated positions and allowed to ride on their parent atoms [C-H 0.93 Å; U_iso_ = 1.2 U_eq_ (parent atom)] or constrained (H atoms of the water molecules). The molecular graphics were prepared using the DIAMOND software [41]. 

CCDC2224951 contains the supplementary crystallographic data for **4c**. These data can be obtained free of charge from The Cambridge Crystallographic Data Centre via www.ccdc.cam.ac.uk/data_request/cif.

#### 3.3.3. IR, NMR and Mass Spectra

IR spectra in the solid state were recorded on a Nicolet iS5 FTIR spectrometer (Thermo Fisher Scientific, Waltham, MA, USA) using an internal reflectance attachment with diamond optical element−attenuated total reflection (ATR) with a 45° angle of incidence. The resolution was 4 cm^−1^; the number of scans was 32. The NMR spectra were recorded in the standard 5 mm sample tubes using an Agilent 400-MR spectrometer (Agilent Technologies, Santa Clara, CA, USA) with operating frequencies of 400.1 MHz (^1^H), and 100.6 MHz (^13^C). Registration of HRMS ESI mass spectra was carried out using an LCMS-IT-TOF instrument (Shimadzu, Japan).

### 3.4. DFT Calculations

DFT calculations were carried out using the ORCA program (B3LYP, def2-TZVP, ECP-60 for Re) [42,43]. The influence of the medium was modeled using the SMD [44] continuum model (water, ε = 78.355).

## 4. Conclusions

The reaction of cyanuric chloride with pyridine takes place at elevated temperatures in the presence of silver nitrate as an activator. As a result of nucleophilic substitution, compounds with two pyridinium rings in its structure can be isolated. It has been found that the formed product can be used as an efficient anion exchanger. Moreover, it can be used for the precipitation of perrhenate anions. The structures of chloride, nitrate and perrhenate salts were established by X-ray analysis. 

## Data Availability

Not applicable.

## Supplementary Materials

The following supporting information can be downloaded at: https://www.mdpi.com/article/10.3390/molecules28041941/s1, Figure S1: X-ray powder patterns; Figures S2–S4: copies of NMR, IR and HRMS spectra, correspondingly; Figures S5–S7: X-ray drawings; Tables S1–S4: crystallographic data for **4a**, **4b** and **4c**.

## Sample Availability

Samples of the compounds are not available from the authors.
