# Supplementary material for: A Promising 1,3,5-Triazine-Based Anion Exchanger for Perrhenate Binding: Crystal Structures of Its Chloride, Nitrate and Perrhenate Salts
Source: Molecules. 2023 Feb 17;28(4):1941. doi: 10.3390/molecules28041941 (PMC9966240; doi:10.3390/molecules28041941)
Supplement: Supplementary file 1 [file molecules-28-01941-s001.zip › molecules-2215888-supplementary.pdf]

*Supplementary Materials*

# A Promising 1,3,5-Triazine-Based Anion Exchanger for Perrhenate Binding: Crystal Structures of Its Chloride, Nitrate and Perrhenate Salts

**Valery N. Zakharov, Pavel S. Lemport, Vladimir V. Chernyshev, Victor A. Tafeenko, Alexandr V. Yatsenko, Yuri A. Ustynyuk, Sergey F. Dunaev, Valentine G. Nenajdenko * and Leonid A. Aslanov ***

Department of Chemistry, Lomonosov Moscow State University, Leninskie Gory 1 bld. 3, Moscow 119991, Russia

* Correspondence: nenajdenko@gmail.com (V.G.N.); aslanov.38@mail.ru (L.A.A.)

## Supplementary Materials

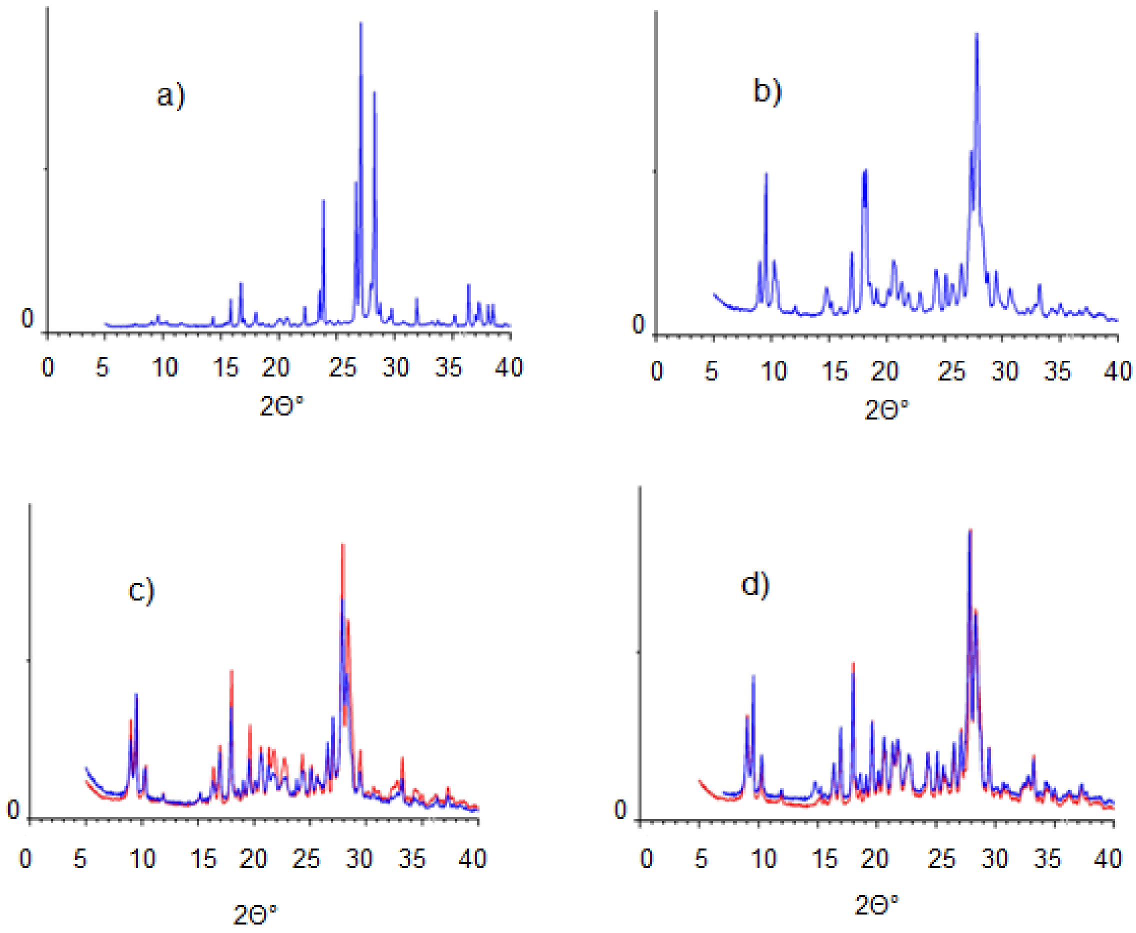


**Figure S1.** X-ray powder patterns for the precipitates that formed in reaction of pyridine and cyanuric chloride after (a) 5 minutes of reaction; b) 10 minutes of reaction; c) 20 minutes of reaction; d) 30 minutes of reaction

**NMR, IR and HRMS spectra**

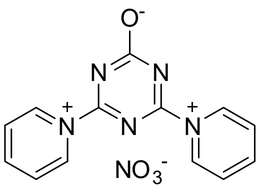


**4,6-di(pyridin-1-ium-1-yl)-1,3,5-triazin-2-olate nitrate 4b.** $^{1}H$ NMR (400 MHz, $D_2O$) δ 10.04 (d, J = 6.3 Hz, 6H), 8.93 (t, J = 7.7 Hz, 3H), 8.33 (t, J = 7.1 Hz, 6H); $^{13}C$ NMR (101 MHz, $D_2O$) δ 169.5, 164.0, 151.3, 140.2, 127.5.

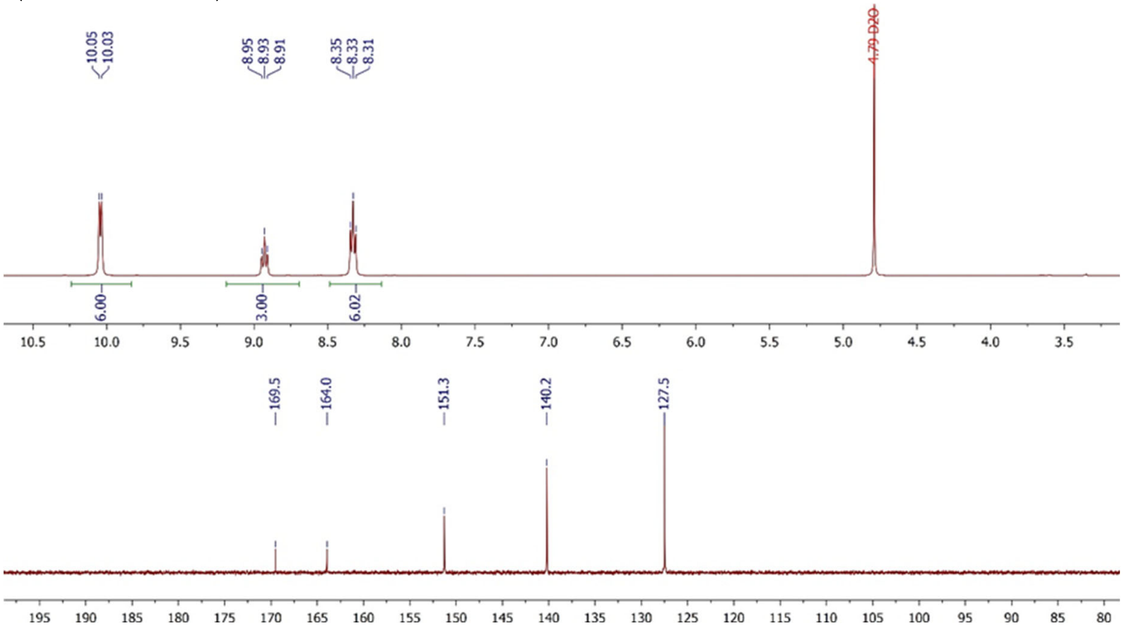


**Figure S2.** NMR $^{1}H$ and $^{13}C$ spectra ($D_2O$, 25°C) of compound **4b**.

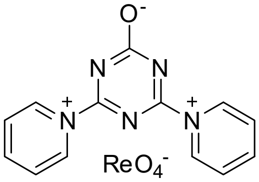


**4,6-di(pyridin-1-ium-1-yl)-1,3,5-triazin-2-olate perrhenate 4c.** Brown solid, m.p. 200-203°C (with decomp.). IR (ν, cm-1) 898 ($ReO_4^-$).

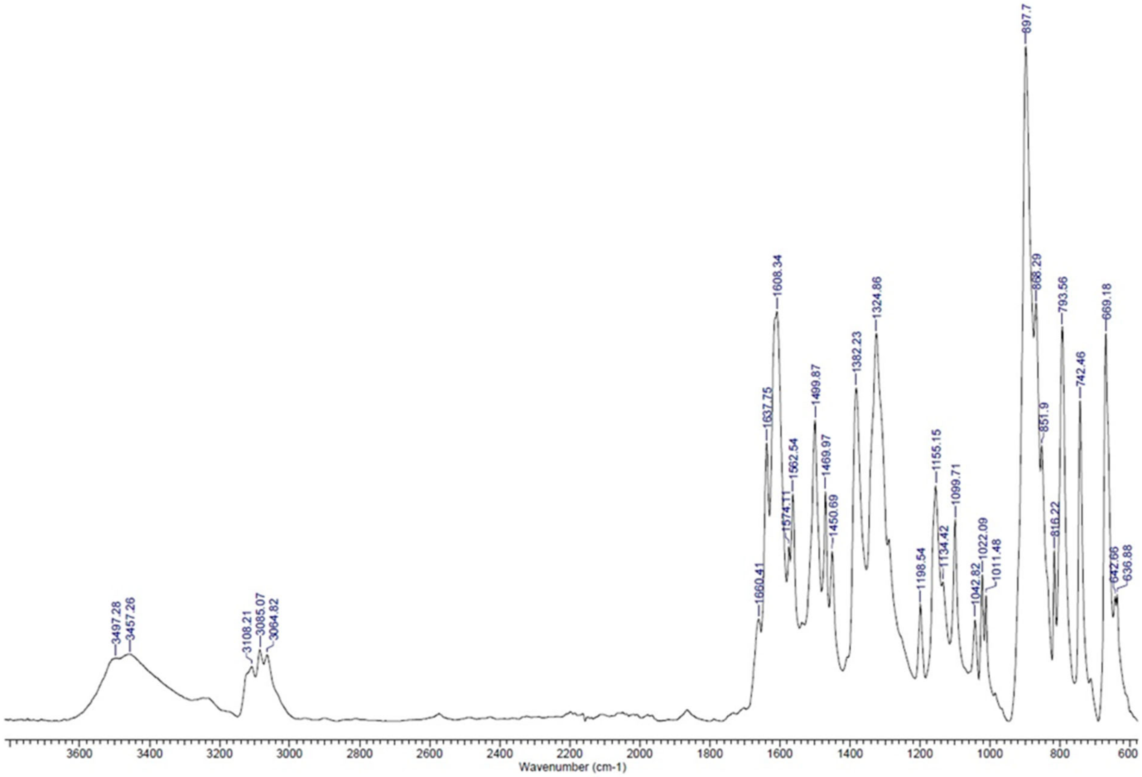

**Figure S3.** Solid-state IR spectrum of compound **4c**.

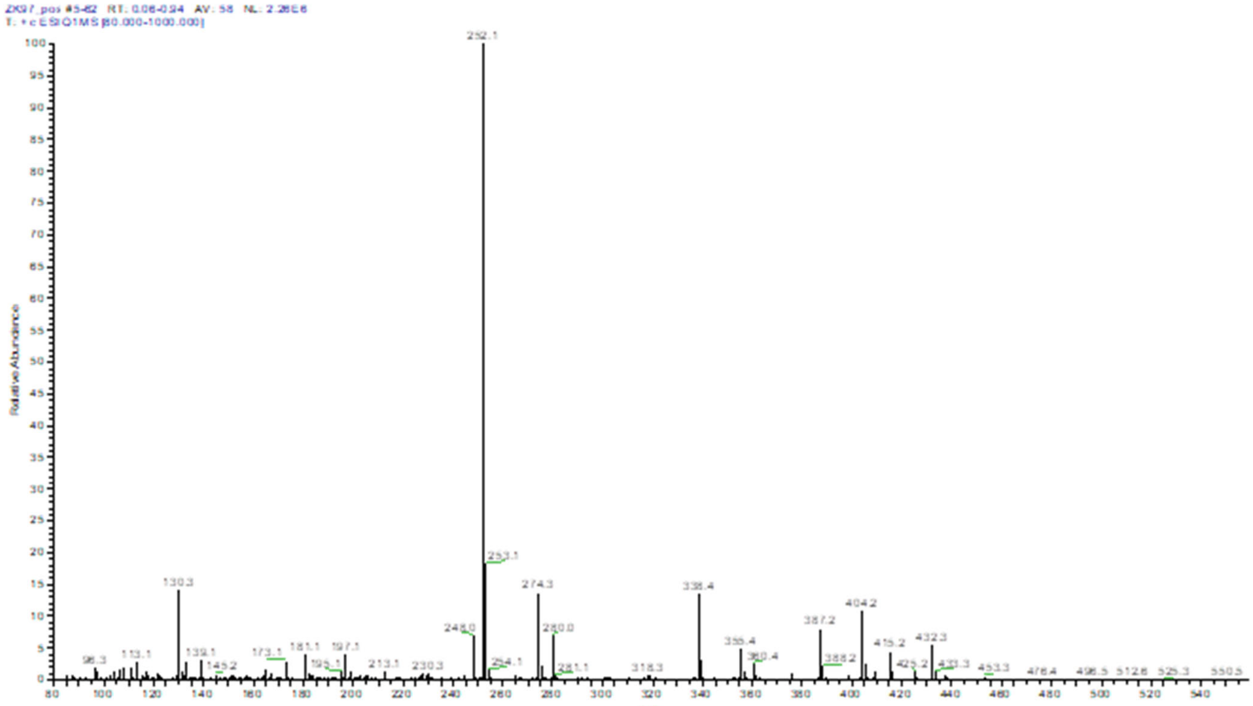

**Figure S4.** Mass spectrum with predominant content of 4,6-dipyridinio-2-oxido-1,3,5-triazine ions.

**Table S1**. Crystal data for **4a**, **4b** and **4c**.

| | **4a** | **4b** | **4c** |
|---|---|---|---|
| empirical formula | $C_{13}H_{10}N_5O^+ \cdot Cl^- \cdot H_2O$ | $C_{13}H_{10}N_5O^+ \cdot NO_3^-$ | $C_{26}H_{26}N_{10}O_{13}Re_2$ |
| $M_r$ | 305.73 | 314.27 | 1058.97 |
| Temperature, K | | | 295(2) |
| crystal system | Monoclinic | Triclinic | Monoclinic |
| space group | *P2₁/c* | *P-1* | C 2/c |
| wavelength, Å | 1.54059 | 1.54059 | 1.54186 |
| unit cell dimensions | | | |
| *a*, Å | 10.1956(11) | 10.1065(11) | 14.5239(3 |
| *b*, Å | 14.9491(14) | 10.2800(12) | 20.3202(6) |
| *c*, Å | 9.3480(9) | 6.6028(7) | 22.6058(6) |
| *α*, ° | 90 | 93.740(13) | 90 |
| *β*, ° | 108.377(19) | 102.313(19) | 102.615(2) |
| *γ*, ° | 90 | 86.345(11) | 90 |
| volume, $Å^3$ | 1352.1(2) | 667.91(13) | 6510.6(3) |
| Z | 4 | 2 | 8 |
| $D_x$ ($Mg\ m^{-3}$) | 1.502 | 1.563 | 2.161 |
| μ, $mm^{-1}$ | 2.630 | 1.027 | 15.045 |
| F(000) | | | 4048 |
| Crystal size | | | 23 x 17 x 14 $mm^3$ |
| $2\theta_{min}$ - $2\theta_{max}$, $\Delta 2\theta$(°) | 7.00 – 75.00, 0.01 | 7.00 – 75.00, 0.01 | |
| no. params/restraints | 97/122 | 101/136 | |
| Data/restraints /parameters | | | 5742 / 11 / 478 |
| $R_p$, $R_{wp}$, $R_{exp}$ | 0.0195, 0.0249, 0.0140* | 0.0209, 0.0271, 0.0151 | |
| Theta range for data collection | | | 4.352 to 66.899° |
| Index ranges | | | -12<=h<=17, -24<=k<=22, -26<=l<=26 |
| Reflections collected | | | 32189 |
| Independent reflections | | | 5742 [R(int) = 0.0901] |
| Completeness to theta = | | | 66.899° 99.2 % |
| Refinement method | | | Full-matrix least-squares on $F^2$ |
| Final R indices [I>2sigma(I)] | | | R1 = 0.0475, wR2 = 0.1014 |
| R indices (all data) | | | R1 = 0.1030, wR2 = 0.1163 |
| Largest diff. peak and hole | | | 3.025 and -1.065 e. $Å^{-3}$ |
| GOF | 1.778 | 1.791 | 0.818 |
| diffractometer | Huber G670 Guinier | Huber G670 Guinier | STOE diffractometer Pilatus 100K detector |
| CCDC code | 2214608 | 2214607 | 2224951 |

* For 4a, which contains 10% of 4b, R-factors were obtained in two-phase refinement.

**Table S2**. Hydrogen-bonding geometry (Å, º) in **4b** crystalline phase.

| D–H…A | D–H | H…A | D…A | D–H…A |
|---|---|---|---|---|
| C12–H12…O3 | 0.95 | 2.24 | 3.076(10) | 147 |
| C8–H8…O4$^{i}$ | 0.95 | 2.44 | 3.145(10) | 131 |
| C9–H9…O2$^{i}$ | 0.95 | 2.20 | 3.101(10) | 158 |
| C10–H10…O1$^{ii}$ | 0.95 | 2.48 | 3.351(10) | 152 |
| C16–H16…O1$^{iii}$ | 0.95 | 2.21 | 3.083(10) | 152 |
| C17–H17…O2$^{iv}$ | 0.95 | 2.33 | 3.163(10) | 146 |

Symmetry codes: (i) x, y–1, z; (ii) x–1, y, z; (iii) x, 1+y, z; (iv) 1+x, y, z.

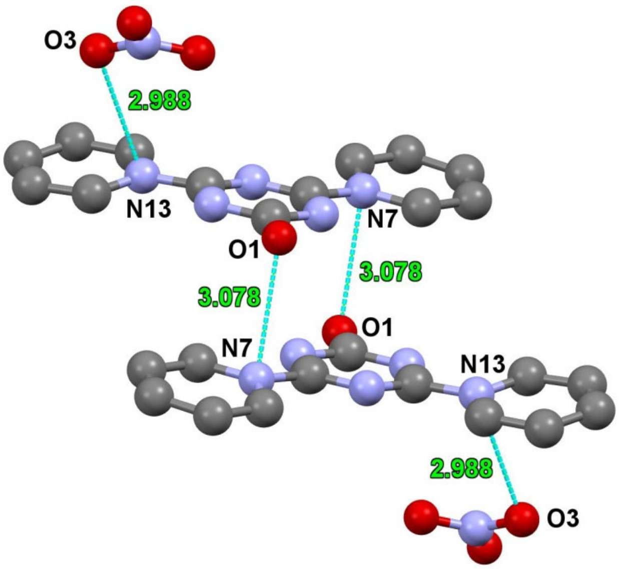


**Figure S5.** Short intermolecular N…O contacts (dotted cyan lines) in the crystal structure **4b**.

**Table S3**. Hydrogen-bonding geometry (Å, º) in **4a** crystalline phase.

| D–H…A | D–H | H…A | D…A | D–H…A |
|---|---|---|---|---|
| O2–H2A…Cl1$^{i}$ | 0.85 | 2.18 | 3.036(6) | 173 |
| O2–H2B…Cl1$^{ii}$ | 0.85 | 2.47 | 3.315(6) | 171 |
| C9–H9…Cl$^{iii}$ | 0.95 | 2.79 | 3.476(9) | 129 |
| C16–H16…Cl1$^{iv}$ | 0.95 | 2.80 | 3.554(10) | 136 |
| C17–H17…Cl1 | 0.95 | 2.69 | 3.560(9) | 152 |
| C14–H14…O1$^{v}$ | 0.95 | 2.17 | 3.071(11) | 158 |
| C12–H12…O1$^{v}$ | 0.95 | 2.23 | 3.150(11) | 162 |
| C10–H10…O2 | 0.95 | 2.34 | 3.114(12) | 139 |

Symmetry codes: (i) 1–x, 1–y, 1–z; (ii) 1–x, y–1/2, 1/2–z; (iii) x–1, 3/2–y, z–3/2; (iv) 2–x, 1–y, 2–z; (5) 1–x, y–1/2, 1/2–z.

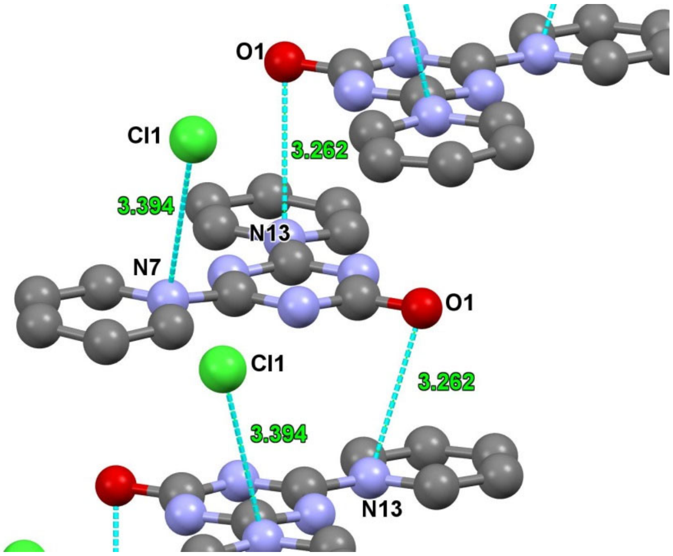


**Figure S6.** Short intermolecular N…O and N…Cl contacts (dotted cyan lines) in the crystal structure **4a**.

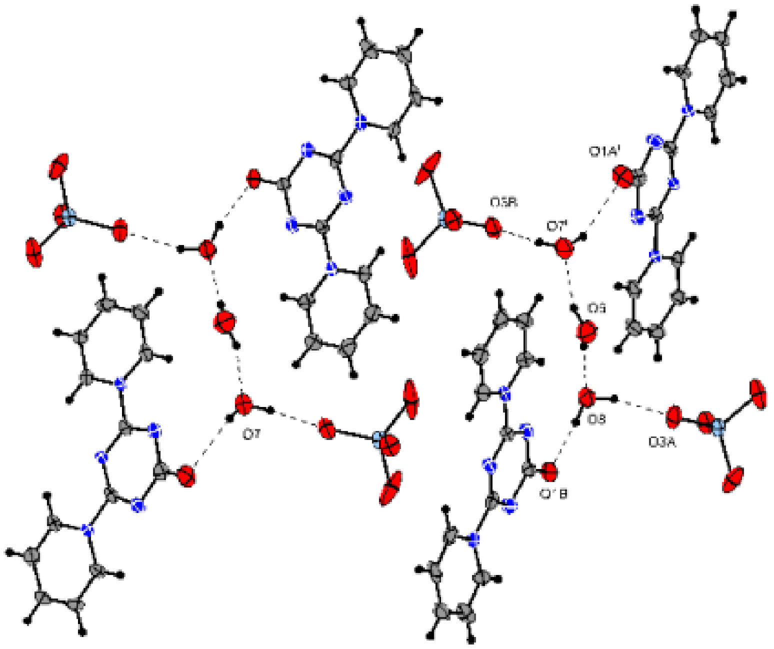


**Figure S7.** Visualization of the hydrogen bonds in the crystal **4c** (code i = -x, y, 0.5-z).

**Table S4** Hydrogen bonds for R1 [Å and angles °].

| | D-H...A | d(D-H)d(H...A) | d(D...A) | <(DHA) |
|---|---|---|---|---|
| C(7A)-H(7A)...O(8)#1 | 0.93 | 2.53 | 3.402(17) | 155.9 |
| C(9A)-H(9A)...O(5B)#2 | 0.93 | 2.47 | 3.094(16) | 124.4 |
| C(10A)-H(10A)...O(5B)#2 | 0.93 | 2.58 | 3.142(14) | 119.8 |
| C(11A)-H(11A)...O(4B)#2 | 0.93 | 2.49 | 3.104(15) | 123.4 |
| C(12A)-H(12A)...O(4B)#2 | 0.93 | 2.57 | 3.145(17) | 120.6 |
| C(14A)-H(14A)...O(8)#3 | 0.93 | 2.63 | 3.536(16) | 165.5 |
| C(15A)-H(15A)...O(1B)#3 | 0.93 | 2.38 | 3.169(16) | 142.2 |
| C(6B)-H(6B)...O(1A)#3 | 0.93 | 2.29 | 3.069(16) | 140.8 |
| C(10B)-H(10B)...O(3A)#4 | 0.93 | 2.64 | 3.405(17) | 139.5 |
| C(11B)-H(11B)...O(3A)#4 | 0.93 | 2.48 | 3.286(16) | 145.6 |
| C(15B)-H(15B)...O(6) | 0.93 | 2.28 | 3.111(18) | 149.1 |
| O(6)-H(61)...O(7)#1 | 0.86(2) | 1.91(6) | 2.733(17) | 160(16) |
| O(6)-H(62)...O(8) | 0.86(2) | 1.97(5) | 2.780(15) | 156(12) |
| O(7)-H(71)...O(1A) | 0.87(2) | 2.30(14) | 2.912(14) | 128(15) |
| O(7)-H(72)...O(3B) | 0.86(2) | 2.01(5) | 2.837(12) | 160(12) |
| O(8)-H(81)...O(3A) | 0.866(19) | 2.00(3) | 2.843(11) | 163(9) |
| O(8)-H(82)...O(1B) | 0.85(2) | 1.97(3) | 2.787(12) | 160(9) |

Symmetry transformations used to generate equivalent atoms:
#1 -x,y,-z+1/2 #2 x,-y+1,z-1/2 #3 -x+1,y,-z+1/2
#4 -x+1/2,-y+1/2,-z+1
